# *Trifolium pratense* L. (red clover) extract and doxorubicin synergistically inhibits proliferation of 4T1 breast cancer in tumor‐bearing BALB/c mice through modulation of apoptosis and increase antioxidant and anti‐inflammatory related pathways

**DOI:** 10.1002/fsn3.1724

**Published:** 2020-07-06

**Authors:** Mohsen Akbaribazm, Mohammad Rasoul Khazaei, Mozafar Khazaei

**Affiliations:** ^1^ Students Research Committee Kermanshah University of Medical Sciences Kermanshah Iran; ^2^ Fertility and Infertility Research Center Health Technology Institute Kermanshah University of Medical Sciences Kermanshah Iran

**Keywords:** 4T1 cell, apoptosis, breast cancer, doxorubicin, isoflavone, *Trifolium pratense* L.

## Abstract

Therapeutic strategies against triple‐negative breast cancer (TNBC) are associated with drug‐induced toxicities. The tropical edible red clover (*Trifolium pratense* L.) is rich in polyphenolic compounds which confer the plant potential anticancer properties. The aim of this study was to investigate the effects of *T. pratense* and doxorubicin (DOX) on the apoptosis and proliferation of 4T1 tumor cells in an allograft model of tumor‐bearing BALB/c mice. Fifty‐six female 4T1‐tumor bearing‐ BALB/c mice were randomly divided into 7 groups (*n* = 8/group) to receive different doses and combinations of DOX and *T. pratense* extract for 35 days. On the 36th day, serum estradiol (E2), IL‐12 and IFN‐γ cytokines, and glutathione peroxidase (GPx) activity were measured. Tumor's ferric reducing antioxidant power (FRAP) and the expressions of apoptosis‐related genes (p53, Bax, Bcl‐2, and caspase‐3) were also evaluated. Immunohistochemical staining for Ki‐67 and p53 were performed. Our results showed that the co‐treatment of DOX and *T. pratense* (100–400 mg/kg) inhibited the proliferation of 4T1 tumor cells in dose‐ and time‐dependent manners. The co‐treatment of DOX and *T. pratense* (especially at the dose of 400 mg/kg) decreased the serum level of E2 (as a stimulant for breast tumor growth) and increased the serum levels of IL‐12 and IFN‐γ along with significant increments in serum GPx and tumor FRAP activities. The co‐administration of DOX and *T. pratense* also decreased the expression of Ki‐67 proliferation marker and increased the number p53 positive (i.e., apoptotic) cells within tumors. This was accompanied with the upregulation of pro‐apoptotic and down‐regulation of antiapoptotic genes. The key findings indicated the synergistic effects of DOX and *T. pratense* against TNBC xenografts.

## Graphical abstract

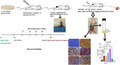

**Highlights**

- *T. pratense* and doxorubicin synergistically, suppressed tumor growth and decreased tumor volume via up‐regulation of apoptosis‐related genes and suppressing estrogen production in 4T1‐tumor bearing BALB/c mice.
- *T. pratense* and doxorubicin synergistically increase survival of tumor‐bearing BALB/c mice.
- *T. pratense* reduces the side effects of doxorubicin via activating antioxidant pathways.

## 1 INTRODUCTION

Breast cancer (BC) is a complex and heterogeneous disease. Regarding pathology, biochemistry, and etiology, 30% of BC tumors are estrogen‐independent. BC is the second leading cause of cancer‐related death. In 2017, about 15% of all the new cases of BC accounted for triple‐negative breast cancer (TNBC) in the United States. In TNBC, neither of human epidermal growth factor receptor 2 (HER2), progesterone receptor, and the estrogen receptor (ER) are expressed (Weigelt & Reis‐Filho, 2009). Therefore, hormone therapy and using monoclonal antibodies (e.g., anti‐HER2 such as trastuzumab) to control and treat these tumors are useless.

The 4T1 is a TNBC cell line which is isolated from BALB/c mice with breast tumors. TNBC is characterized by poor prognosis compared with other types of BC. It has a higher tendency for metastasis to the brain, lung, liver, lymph nodes, and bone marrow (Bazm, Naseri, & Khazaei, 2018). Recently, using medicinal herbs alongside with effective chemotherapeutics (which usually have numerous side effects) has been regarded as one of the most promising approaches in BC treatment and management (Shahid, 2013). Therefore, developing novel anticancer agents with synergistic effects and fewer toxicity from natural products and organisms, especially plants, has been considered as an alternative and cost‐effective therapeutic modality.

Anthracyclines are anticancer drugs including doxorubicin (DOX), which are derived from *Streptomyces* bacteria, especially *S. Caesius* and *S. Peucetius* strains. These drugs, which induce the formation of free radicals and inhibit the intercalation of topoisomerase II and DNA, have delivered therapeutic success rates from 40%–50% to 60%–80% (in synergy with other drugs such as Curcumin, Quercetin, and Ocotillol) in BC. The synergism of these drugs with other therapeutic agents can effectively induce a variety of anticancer pathways and improve their safety profile by decreasing their side effects (i.e., cardiotoxicity, thrombocytopenia, stomatitis, acute nausea, gastrointestinal disturbances, bone marrow aplasia, and vomiting; Nadas & Sun, 2006; Lv et al., 2016).

Nowadays, using herbs showing anticancer properties in combination with other therapeutic compounds (e.g., cell cycle regulators, growth factor‐related molecules, hormone analogs, and cellular stress‐reducing compounds) provides an interesting research field in commercial pharmaceutics (Block et al., 2008; Shahriari, Hemmati, Zangeneh, & Zangeneh, 2019; Zangeneh, Ghaneialvar, et al., 2019). Isoflavones and flavonoids are polyphenolic compounds that can be used either alone or in combination with other drugs to treat BC. These compounds are more commonly used in developing countries (Asian and Latin American countries) where the incidence of this cancer is relatively lower than in developed countries (Lee et al., 1991; Zangeneh, Joshani, Zangeneh, & Miri, 2019).

Clover (*Trifolium pratense* L.), a member of the fabaceae family, has been used for medicinal purposes due to its high content of important flavonoids (quercetin, kaempferol, and apigenin) and isoflavones (formononetin, biochanin A, genistein, and daidzein; Ellison, Liston, Steiner, Williams, & Taylor, 2006). The extract of *T. pratense* is used as an expectorant, analgesic, anticoagulant, antiseptic, and febrifuge agent in traditional medicine. Furthermore, the plant extract has been applied to improve or treat menopausal symptoms, blood sugar and lipids, cardiovascular and osteoporotic markers, fertility, and nervous system disorders (Booth et al., 2006; Kroyer, 2004). The isoflavones of *T. pratense* are analogues of estrogen and bind to α and β‐ERs (ERα and ERβ) in various tissues including the breast and endometrium (Zhang et al., 2010). Isoflavones have antiestrogenic roles during premenopausal period and represent weak estrogenic activities (such as dilating the breast ducts of mammary glands) after menopause (Adlercreutz, 2002; Mahdavi et al., 2019).

Many studies have reported that isoflavones can suppress the growth of BC tumor cells in vitro and in vivo (Ahmeda & Zangeneh, 2020). An in vivo study on nude mice bearing MCF‐7 and MDA‐MB‐231 cell lines xenografts demonstrated that genistein inhibited the tumor growth and invasive behavior of BC cells by downregulating matrix mettaloproteinase‐9 (Zhou et al., 2014). Daidzein also inhibited the proliferation of BC cells and induced apoptosis by increasing the Bax/Bcl‐2 ratio and inhibiting inflammatory cytokines (Thangapazham, Passi, & Maheshwari, 2007). A diet containing isoflavones also suppressed BC growth via inducing apoptotic pathways (i.e., up‐regulating Bax, Caspase‐3, and p53 genes), blocking and reducing estrogen production, and decreasing reactive oxygen species (ROS) formation (Uifălean, Schneider, Ionescu, Lalk, & Iuga, 2016). Considering the difficulty of BC treatment and a need to develop new therapeutic strategies, the aim of the present study was to investigate the synergistic anticancer effects of *T. pratense* extract with DOX against BC in 4T1‐tumor bearing BALB/c mice.

## 2 MATERIALS AND METHODS

### 2.1 Plant collection and extract preparation

After preparing the seeds (Karaj Seed and Plant Improvement Institute) and the plant of *T. pratense* (the Research Farm of Kermanshah University of Medical Sciences), they were identified and authenticated by a botanist and taxonomist (Agricultural Research, Education, Extension Organization; voucher no: KPC/ Kulubara‐1274). Its fresh leaves were harvested and dried in shade at room temperature (25 ± 2°C). The dried leaves were grounded and mixed with ethyl alcohol (70%) for 2 days. Then, the solution was filtered through filter papers (Whatman no. 42, Millipore Cat no. 1,442,125). The extract was condensed after the evaporation of alcohol and stored at 4°C (Bazm, Khazaei, Ghanbari, & Naseri, 2018).

### 2.2 BC induction

4T1 mouse mammary carcinoma cells (ATCC CRL‐2539) were obtained from Pastor Institute cell bank (Tehran, Iran). The cells were cultured in RPMI‐1640 medium supplemented with nonessential amino acids, 1% penicillin‐streptomycin, and 10% fetal bovine serum (Sigma). The cultures were maintained in a humidified atmosphere with 5% CO_2_ at 37°C. Then, the cultured cells (>85% viability) were harvested using trypsin‐EDTA (Invitrogen). Finally, 1 × 10^6^ cells were diluted in PBS and injected subcutaneously into the right mammary fat pad of female BALB/c mice aged 5–6 weeks old (Pastor Institute). The tumors grew and became palpable after 10 days (70% incidence rate; Khazaei & Pazhouhi, 2019).

### 2.3 Animals and treatments

Fifty‐six 4T1‐tumor bearing mice were categorized into seven groups (*n* = 8/group) for Kaplan–Meier survival curve and other analyses. Eight normal mice were selected for stereological and histopathological studies (*N* = normal saline group). All the animals housed at 23 ± 2°C and 50 ± 10% humidity under 12‐hr light/dark cycles and free access to standard pellet and water. The protocols were approved by the Ethics Committee of Kermanshah University of Medical Sciences (Ethic code: IR.KUMS.REC.1398.359) and performed in line with the guidelines of the Animal Ethics Committee (NIH Publication 80–23, 1996).

### 2.4 Study groups

The study groups included N: normal mice (without 4T1 tumors): the mice received 0.5 ml distilled water (DW) by gavage, t400t: received 400 mg/kg of the *T. pratense* extract, t100, t200, and t400: received 100, 200, and 400 mg/kg of the extract plus a single dose of 5 mg/kg DOX (intravenous, i.v.), C+: received a single dose of 5 mg/kg DOX (i.v.), and C−: 4T1 mice receiving 0.5 ml DW.

**FIGURE 1:**
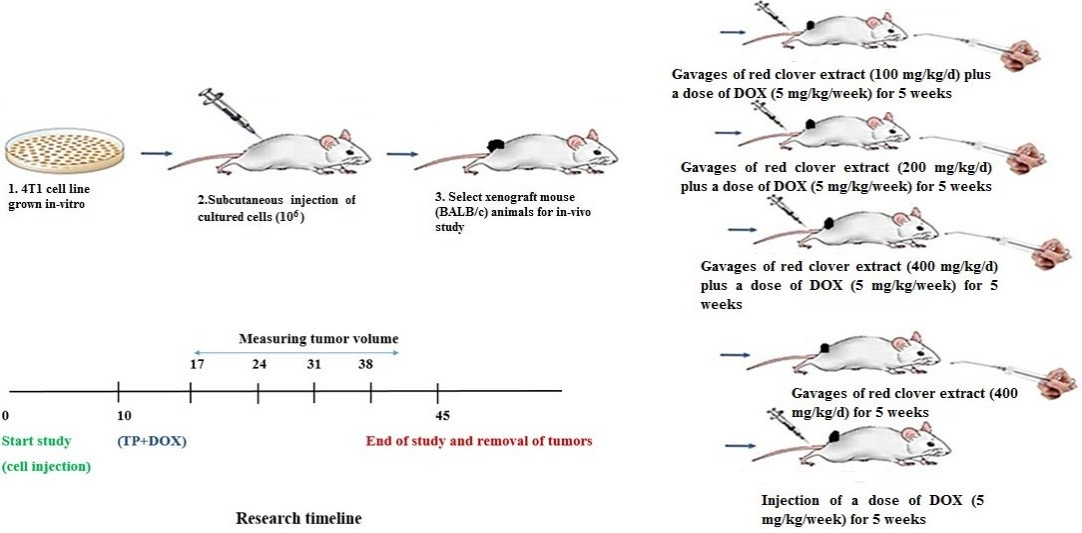
Research time line and experimental design. DOX, doxorubicin

The mice were fed with the extract (dissolved in 0.5 ml DW) by gavage for 6 days a week and 5 consecutive weeks (El‐Gendy, 2012). Normal saline (N) and *T. pratense* extract were administrated intravenously (IV). The weights of the animals and tumor sizes were regularly measured and recorded in all the groups every week. At the end of each week, the positive control and synergistic groups received a single dose of DOX (i.v., dissolved in 0.5 ml DW; Figure 1).

### 2.5 Tumor size and survival assay

Tumor development, tumors size (with vernier calipers), survival rate, and body weight were measured every day. Tumor volume was calculated using the large (length) and small (width) diameters of the tumors using the following formula:

$$\text{Volume}\;\left({\text{mm}}^{3}\right)=\left[{\left(\text{width}\right)}^{2}\times \text{length}\right]/2.$$

**FIGURE 2:**
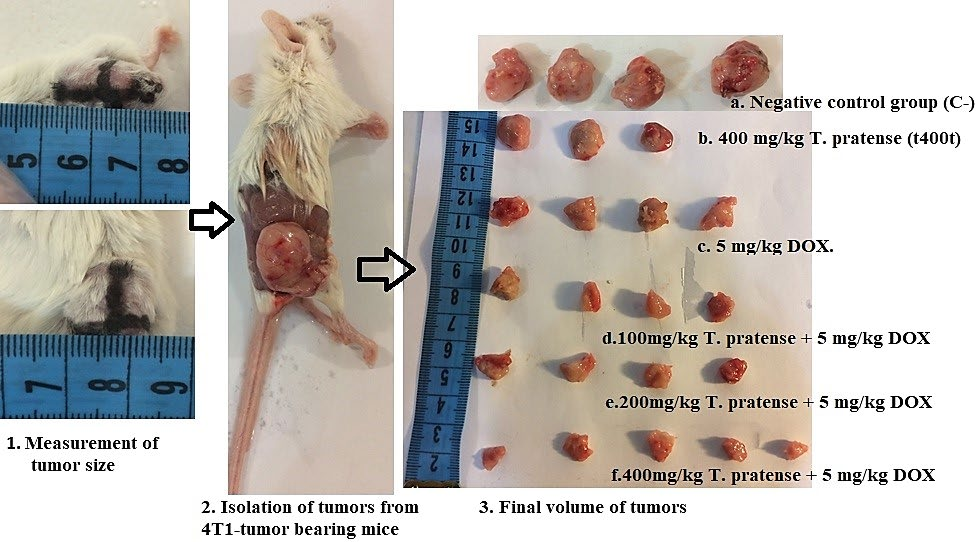
4T1‐tumor bearing mice and tumor samples in negative control group (C−), doxorubicin (DOX) group (5 mg/kg DOX), 400 mg/kg *Trifolium pratense* and co‐treatment groups (100, 200, and 400 mg/kg *T. pratense* plus 5 mg/kg DOX)

To evaluate the survival rate, the number of deaths in each group was recorded daily until the end of the study (the day 45th after tumor induction). The data were then analyzed by Kaplan–Meier test (Taheri, Dinarvand, Ahadi, Khorramizadeh, & Atyabi, 2012; Figure 2).

### 2.6 Tumors histopathology

#### 2.6.1 Stereological histopathology

**FIGURE 3:**
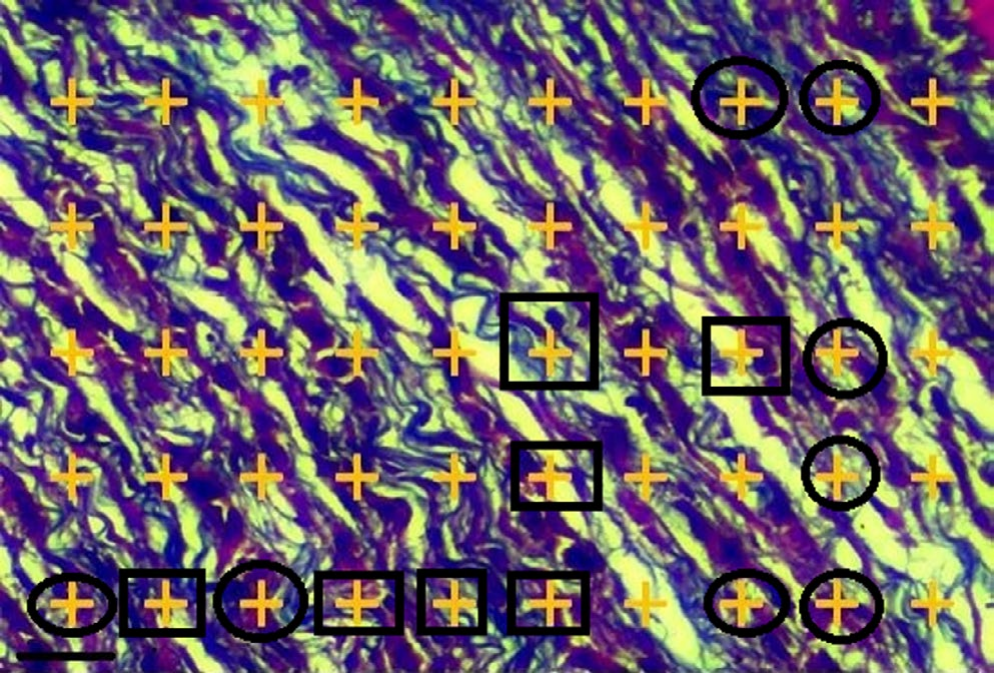
Point probe (50 points) for calculating the histopathological changes of tumor tissue. The total number of points located on each structure (circle [points on the cell] and square [points on the collagen fiber]) is counted in each field (10 field/tumor) of view and is compared with the total number of points in the volume density formula: *V*_v_ = ∑*P*_4T1 cell_/∑*P*_collagen fiber_ (Masson's trichrome scale bar = 30 μm, ×400)

**FIGURE 4:**
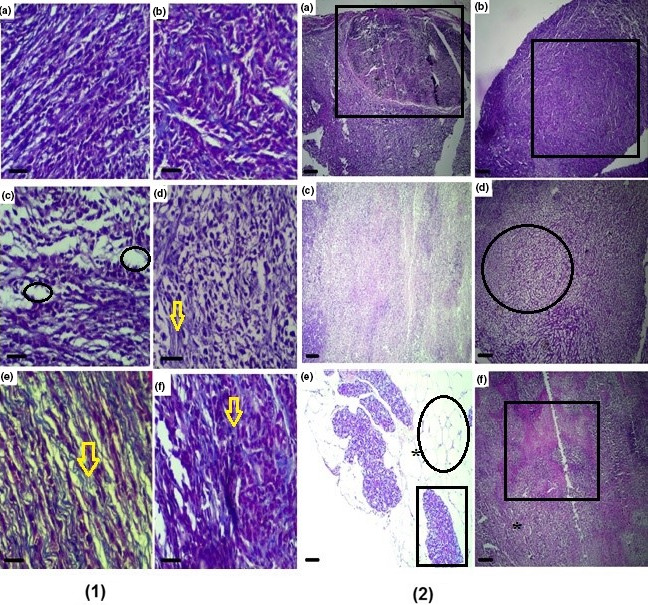
Evaluation of histopathological changes in negative control (a), positive control (doxorubicin, b) and the extract groups [t100 (c), t200 (d) and t400 (e)] and t400t (f) according to stereological principles discussed in material methods section and also in Figure 3. Tumor space (squares), decrease in tumor cell nucleus density and replacement with parenchyma (circle) and increased intercellular fibers synthesis (yellow arrow) [(1) Masson's trichrome: scale bar = 30 μm, ×400 and (2) hematoxylin and eosin: scale bar = 300 μm, ×40] staining

Tumor tissues were removed and fixed in 10% formalin for 72 hr. The final volume of tumor tissues was measured. Then, paraffin blocks were prepared from the processed tumor tissues, and finally 5‐μm sections were prepared from the blocks (Leica Microsystems). The tissue sections were stained by the Masson's trichrome and hematoxylin and eosin. Finally, the cells and intercellular matrix (including collagen fibers) were evaluated using a Nicon light microscopy equipped with a KEcam (KEcam Technologies), point probe (50 points), and Top view software (Version 3.7). The numbers of points (representing 4T1 tumor cell nucleus) and collagen fibers in each counting area were determined to calculate a numerical density ratio (tumor cell nucleus/collagen fibers) in each tumor tissue by evaluating at least 10 field/tumor at the × 400 magnification. The density ratio was expressed as mean ± *SD*. With increasing apoptosis in tumor cells, collagen fibers (as scar tissues) replaced the 4T1 cells; therefore, a higher numerical density ratio indicated lower cellular apoptosis (Akbari, Goodarzi, & Tavafi, 2017; Figures 3 and 4).

#### 2.6.2 Immunohistochemical assay

Cellular proliferation and apoptotic index in tumors were determined by immunohistochemical (IHC) for Ki‐67 and p53 markers. In BC tumors, the expressions of Ki‐67 protein, as an indicator of cellular proliferation, and p53, as an indicator of apoptosis, are important to determine the fate of tumor cells. Samples were extracted from 10% formalin within the paraffin blocks to prepare tissue sections (5‐µm thickness) on glass slides covered with 3‐Triethoxysilyl‐propylamine. The slides were incubated in 65°C overnight. All the slides were then deparaffinized, rehydrated, and incubated with EDTA‐Tris buffer (0.4 g EDTA + 2 g tris dissolved in 1 L of distilled water, pH = 9) at 95°C for 20 min for antibody retrieval. Finally, the slides were incubated with 5% BSA to block the endogenous peroxidase. After that, they were incubated in 3% H_2_O_2_ and then washed in PBS twice for 3 min.

The slides were initially incubated with biotinylated rabbit antimouse IgG antibody (Biolegend) at room temperature for 60 min, then with streptavidin‐horseradish peroxidase (i.e., evasion solution) for 30 min, and finally with 1, 3‐diaminobenzidine tetrahydrochloride for 10 min. Subsequently, the slides were counterstained with hematoxylin. At each step, the slides were washed with Tris buffer (0.6 g tris + 8 g NaCl tris dissolved in 1 L of distilled water, pH = 7). A light microscope (Olympus IX71 microscope) equipped with the KEcam (KEcam Technologies), Top view software (Version 3.7), and a point prob was used to analyze the tissues. The data were presented as the percent of Ki‐67‐ and p53‐positive/total points in the point prob. In this method, the ratio of circle points (i.e., Ki‐67 and p53 positive cells)/square points (i.e., Ki‐67 and p53 negative cells) × 100 was calculated over 10 random fields at × 400 magnification. The data were reported as mean ± *SD* (Laurinavicius et al., 2012).

#### 2.6.3 Hormones and cytokines assays

On the day 45th, all the animals were sacrificed with intraperitoneal injection of ketamine (40 mg/ kg), and blood samples were taken from the heart. The sera were isolated (12,000 *g* for 15 min) and stored at −20°C. The serum levels of IL‐12 (p70) and IFN‐γ cytokines (IL‐12, Cat No: 577,004; IFN‐γ, Cat No: 430,807; BioLegend, San Diego, USA) and β‐estradiol (Cat No: ab23449; Abcam) were measured by commercial enzyme‐linked ELISA kits following the manufacturers’ instructions. Briefly, a 96‐well plate was precoated with the primary antibody (overnight at 4°C). After three times of washing with PBS, the samples were incubated with blocking bovine serum albumin for 1 hr. After preparing a standard dilution, the samples were added to the plates and incubated with the diluted detection antibody for 30 min. By the emergence of a yellow color after the substrate reaction (TMB substrate), the optical densities (ODs) were read at 450 and 570 nm by a microplate reader (Biotek Instruments). The serum levels of E2 were reported based on pg/dl (Akbari Bazm, Goodarzi, Shahrokhi & Khazaei, 2020).

#### 2.6.4 Oxidative stress evaluation

Glutathione peroxidase (GPx) activity was assessed with a commercial ELISA kit (Randox Cat No: RS504; Randox Laboratories Ltd.) according to the manufacturer's instructions. This kit indirectly measured GPx activity based on the oxidation of glutathione (i.e., the transformation of GSH to GSSG) which is catalyzed by GPx. The oxidized (GSSG) glutathione is then recycled to GSH by glutathione reductase by consuming NADPH as a reducing power. After the oxidation of NADPH to NADP, the rate of NADPH reduction (corresponding to the GPx activity) was measured by reading the OD at 340 nm and expressed as U/ml of serum.

The total antioxidant capacity (TAC) of serum was measured by the ferric ion reducing antioxidant power (FRAP) method described by Ghanbari, Nejati, and Khazaei (2016). In this method, 90 μl of the FRAP reagent [a mixture of 50 ml of 0.1 M acetate buffer [pH 3.6] in 5 ml of 10 mmol/L 2,4,6‐tripyridyl‐s‐triazine [TPTZ], 5 ml of 20 mmol/L FeCl_3,_ and 0.6 ml HCl 40 mmol/L) was added to 200 μl of tumor tissue homogenates and incubated at 37°C for 4 min. In the FRAP assay, antioxidants can reduce Fe^3+^‐TPTZ salt to its blue colored Fe^2+^‐TPTZ form. Finally, the mixture was centrifuged at 15,000 g for 10 min, and the absorbance of the supernatant was read at 593 nm against a standard curve of FeSO_4_ (0–1,000 μM). The results were reported as µmol/mg protein (Ghanbari et al., 2016).

### 2.7 Quantitative real‐time PCR

#### 2.7.1 RNA extraction and cDNA synthesis

Total RNA was extracted with FavorPrep^™^ Tissue Total RNA Mini Kit (Cat No: FATRK 001; Favorgen Biotech Corp, PingTung Agricultural Biotechnology Park). First, 40 mg of tumor samples was homogenized on ice and transferred into the RNA extraction solution (Trizol) according to the manufacturer's protocol. The remaining cellular debris were removed by centrifuging (12,000 g for 10 min). The quality and purity of the extracted RNA were determined by electrophoresis on 2% agarose gel and determining the *E* = *A*_260_/*A*_280_ and *A*_260_/*A*_230_ ratios by a Nano‐Drop spectrophotometer (Bio‐TeK). The cDNA was synthesized using 1,000 ng of the extracted RNA (BioFact™ cDNA synthesis kit, Cat. No. BR123‐R10k; BioFact^™^ RT Series). The reaction mixture was prepared by admixing 1 µl of Random Hexamer primer, 10 µl of 5× reverse transcription (RT) buffer, 1 µl of oligo‐d (T), and 1 µg of total RNA and reached to a final volume of 20 µl by adding RNase‐free water. The RT reaction was carried out at 95°C (2 min), and then 60°C (30 s), and for the enzymatic reaction; the RT mixture was incubated at 74°C (4 min). The cDNA template was stored at −20°C until use (Akbari Bazm, Khazaei, & Khazaei, 2020).

#### 2.7.2 Real‐Time qRT‐PCR assay

The mRNA levels of p53, caspase 3, Bax, and Bcl‐2 were determined using High ROX BioFact™ 2× Real‐Time PCR Smart mix SYBR Green PCR master mix by a Real‐Time PCR light cycler device (StepOne^™^ Real‐Time PCR System). The primer sequences for Caspase‐3 were as follows: 5′‐CTGGACTGTGGCATTGAGAC‐3′ (forward) and 5′‐GCAAAGGGACTGGATGAACC‐3′ (reverse); for Bcl‐2:5′‐TGGGATGCCTTTGTGGAACT‐3′ (forward), and 5′‐GAGACAGCCAGGAGAAATCA‐3′ (reverse); for p53: 5′‐AGAGACCGCCGTACAGAAGA‐3′ (forward), and 5′‐GCATGGGCATCCTTTAACTC‐3′ (reverse); and for Bax: 5′‐CCGGCGAATTGGAGATGAACT‐3′ (forward), and 5′‐CCAGCCCATGATGGTTCTGAT‐3′ (reverse). The primer sequences for glyceraldehyde‐3‐phosphate dehydrogenase (GAPDH) were as 5′‐AACTTTGGCATTGTGGAAGG‐3′ (forward), and 5′‐ACACATTGGGGGTAGGAACA‐3′ (reverse).

All the primers were designed by Oligo software, and the sequences were blasted in the NCBI database. GAPDH was used as a housekeeping gene. The qRT‐PCR reaction mixture was prepared by blending 10 μl of the SYBR Premix ExTaqII, 1 μl of the forward and reverse primers, and 1 μl cDNA template (a total reaction volume of 20 μl). PCR cycles consisted of 2 min at 50°C, 4 min at 95°C (polymerase activation) followed by 45 cycles of 30 s at 95°C, (denaturation), 1 min at 60°C (annealing), and 1 min at 72°C (extension). Melting curve analysis was carried out between 60 and 95°C (1°C increments) for 5 s at each step. The qRT‐PCR reactions were carried out in duplicate, and GAPDH served as an intrinsic control for each sample. The threshold cycle (CT) of each gene was normalized against GAPDH as the housekeeping gene. The mRNA expression levels were measured by using the Ct (2^‐ΔΔt^) method.

$$\Delta \Delta \text{CT}=\left[{\left({\text{mCT}}_{\text{target}}-{\text{mCT}}_{\text{reference}}\right)}_{\text{test sample}}-{\left({\text{mCT}}_{\text{target}}-{\text{mCT}}_{\text{reference}}\right)}_{\text{control sample}}\right].$$

Finally, the expression levels of target mRNAs were determined as 2−^∆∆CT^ (Akbari Bazm et al., 2020).

### 2.8 Statistical analysis

All statistical analyses except for the survival analysis were performed in SPSS 16.0 applying the one‐way ANOVA test followed by the post hoc Duncan test at the significance level of *p* < .05. The normality of the data was determined by Kolmogorov–Smirnov test (*p* > .05). The survival rate was analyzed by the Kaplan–Meier test via GraphPad Prism statistical software package version 8. All the results were expressed as mean ± *SD*.

## 3 RESULTS

### 3.1 Body weight, tumors size, and survival rate

**FIGURE 5:**
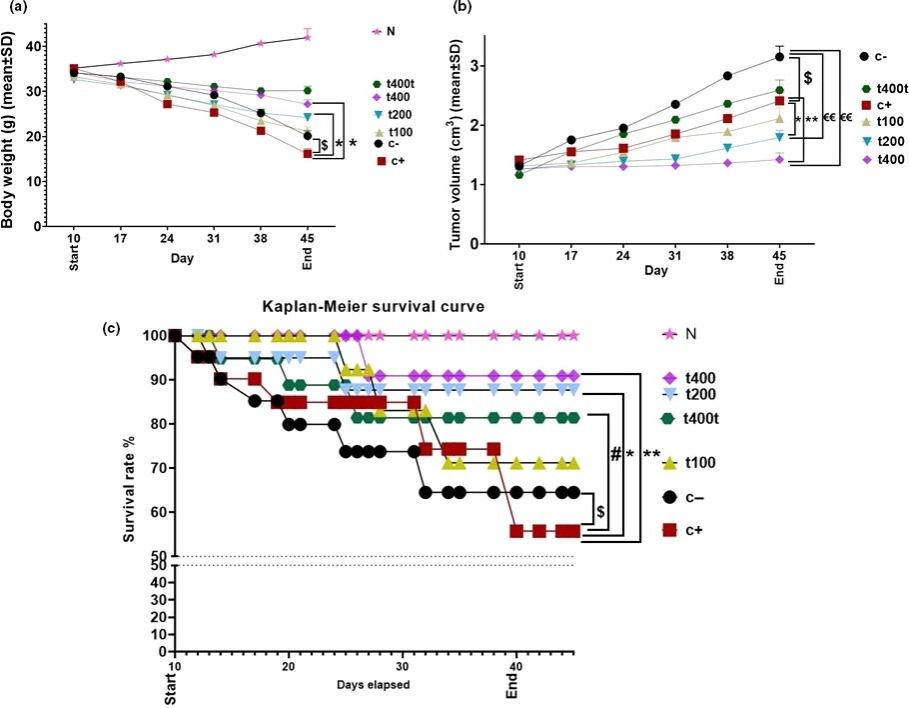
(a) Body weight (g), (b) Volume of tumors (cm^3^) and (c) Kaplan–Meier survival curve in negative control (C−), positive control (doxorubicin [DOX]), the extract groups (t100, t200, t400) and t400t (*M* ± *SD*; mean difference between groups compared by using one‐way ANOVA test). ^$^(*p* < .05) statistically significant between DOX and negative control group, ^#^(*p* < .05) statistically significant between t400t and DOX, ^€€^(*p* < .01) statistically significant between t200 and t400 with negative control (C−), *(*p* < .05) and **(*p* < .01) statistically significant between treatment and DOX group

Body weights were measured on the days of 10, 17, 24, 31, 38, and 45 after tumor induction. The results showed that the induction of 4T1 tumors significantly (*p* = .011) decreased the animals’ weights in all the groups at the end of study (i.e., the day 45) compared with normal group (N) and mean weights recorded at the day 10th (*p* = .016). Furthermore, the mean weight of animals was significantly lower in the DOX compared with the C− group (*p* = .015). However, body weights were significantly higher in the DOX + t400 (*p* = .019) and t400t (*p* = .01) compared with the DOX group (Figure 5a).

Also, tumor volumes were recorded on the days of 10, 17, 24, 31, 38, and 45. In all the groups, the tumors significantly (*p* = .011) increased in size compared with the first day of volume measurement (i.e., day 10). DOX significantly reduced tumor volume compared with the negative control group. On the day 45, tumor volumes were significantly lower in the DOX + t200 (*p* = .016) and DOX + t400 (*p* = .007) compared with the DOX and C− groups (*p* = .008 and *p* = .002, respectively; Figure 5b).

Kaplan–Meier survival analysis showed that at the end of the study, the number of survived mice decreased in all the groups. The survival of mice in the DOX group was significantly lower than in the C− group (*p* = .021). However, the numbers of survived mice in the t400t (*p* = .029), DOX + t200 (*p* = .01), and DOX + t400 (*p* = .008) groups were significantly higher than the DOX group (Figure 5c).

### 3.2 Histopathological findings

Based on histopathological evaluations with stereological principals, the numerical density of 4T1 cells/collagen fiber was significantly higher in tumor‐bearing C− than normal mice (*p* = .015). In the DOX + t200 (*p* = .012) and DOX + t400 (*p* = .008) groups, the numerical densities of tumor cells were significantly lower than the C− group (Table 1; Figure 4).

**TABLE 1:** Histopathological examination of experimental groups. Based on numerical density ratio of the tumor cell nucleus/collagen fibers in each tumor tissue (10 field/tumor)

Groups	Histopathological examination in tumor (*M* ± *SD*)
Normal group	0.3 ± 0.016
Negative control (C−)	2.85 ± 0.31^&^
Positive control (C+)	2.31 ± 0.6
DOX + *T. pratense* 100 mg/kg	2.11 ± 0.3
DOX + *T. pratense* 200 mg/kg	1.71 ± 0.42*
DOX + *T. pratense* 400 mg/kg	0.82 ± 0.2**
*T. pratense* 400 mg/kg	2.41 ± 0.75

Note

The effect of doxorubicin (DOX) and *Trifolium pratense* on histopathological examination of tumor tissue in normal tissue, negative control (C−), positive control (DOX) and the extract groups [t100, t200, and t400] and t400t (*M* ± *SD*; a higher score indicates low cell apoptosis). Mean difference between groups compared by using one‐way ANOVA test; ^&^(*p* < .05) statistically significant between negative and normal control group, *(*p* < .05) and **(*p* < .01) statistically significant between treatment and C− group.

### 3.3 Serum cytokine levels

**FIGURE 6:**
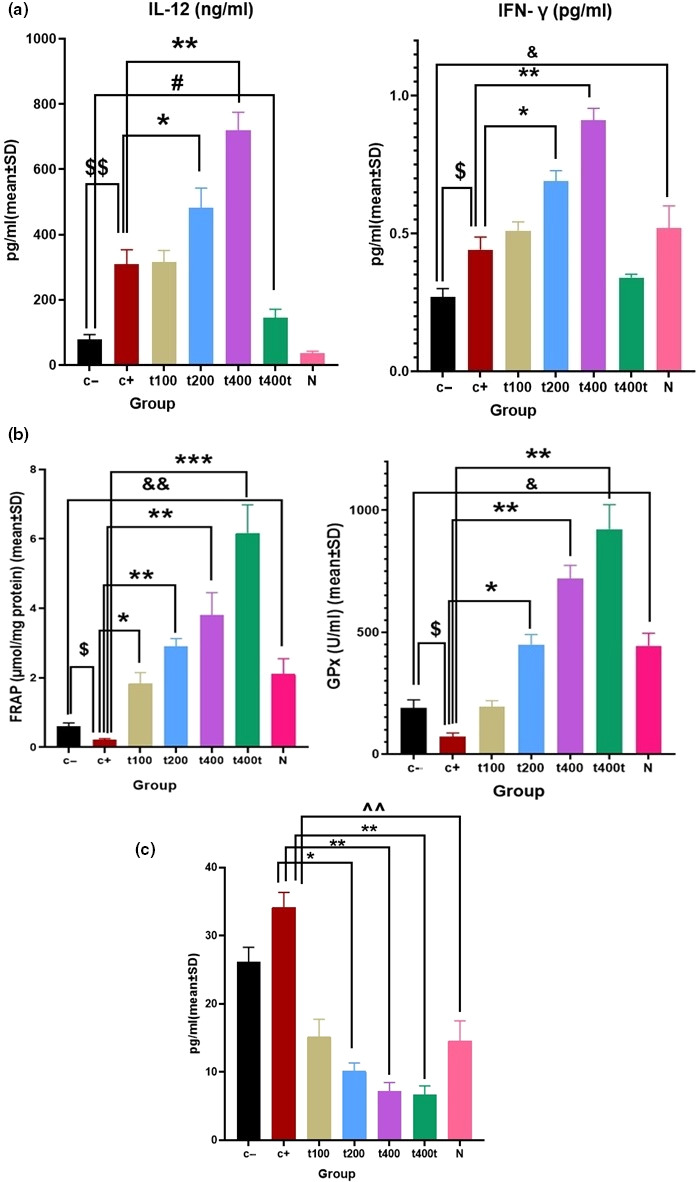
(a) Comparison of serum cytokine levels of IFN‐γ (pg/ml) and IL‐12 (ng/ml) (b) tumors FRAP levels (µmol/mg protein) and serum GPx activity (U/ml) and (c) serum estradiol levels (pg/ml) in negative control (C−), positive control (doxorubicin [DOX]), the extract groups (t100, t200 and t400) and t400t (*M* ± *SD*; mean difference between groups compared by using one‐way ANOVA test). ^$^(*p* < .05) and ^$$^(*p* < .01) statistically significant between DOX and negative control group, ^#^(*p* < .01) statistically significant between t400 and C− group, *(*p* < .05), **(*p* < .01) and ***(*p* < .001) statistically significant between treatment and DOX group, ^&^(*p* < .05) and ^&&^(*p* < .01) statistically significant between C− and normal group and ^^(*p* < .01) statistically significant between DOX and normal group

The serum level of IFN‐γ significantly (*p* = .018) decreased in the C− compared with the normal group. DOX administration significantly increased the serum levels of IL‐12 (*p* = .006) and IFN‐γ (*p* = .037) compared with the C− group. In the animals receiving the plant extract, there were significant elevations in serum IFN‐γ and IL‐12 in the DOX + t200 (*p* = .021 and *p* = .011, respectively) and DOX + t400 (*p* = .007 and *p* = .002, respectively) groups. Also, a significant reduction was noted in the serum level of IL‐12 in the t400t compared with the DOX group (*p* = .029, Figure 6a).

### 3.4 TAC and GPx levels

Tumor tissue FRAP level (0.22 ± 0.026 µmol/mg protein, *p* = .009) and serum GPx activity (103.91 ± 20.11 U/ml, *p* = .031) were significantly lower in the DOX compared with the C− (0.61 ± 0.09 µmol/mg and 175.91 ± 29.11 U/ml, respectively) group. The FRAP level (3.87 ± 0.51 µmol/mg, *p* = .009) and serum GPx activity (724.19 ± 61.11 U/ml, *p* = .006) in the DOX + t400 and DOX + t200 (1.84 ± 0.39 µmol/mg; *p* = .009, and 481.29 ± 41.18 U/ml; *p* = .012, respectively) groups also significantly increased compared with the DOX group. The 400 mg/kg dose of *T. pratense* extract significantly increased the mean level of FRAP (6.17 ± 0.84 µmol/mg, *p* = .0002) and serum GPx activity (864.41 ± 63.11 U/ml, *p* = .001) of tumor tissues in comparison with the DOX group. In the normal (N) group, FRAP (2.18 ± 0.29 µmol/mg, *p* = .018) and GPx (459.18 ± 35.17 U/ml, *p* = .011) levels were significantly lower compared with untreated 4T1‐bearing mice (C−group; Figure 6b).

### 3.5 Serum estradiol level

The serum levels of estradiol (pg/ml) in the DOX + t200 (10.16 ± 1.17 pg/ml, *p* = .016), DOX + t400 (7.14 ± 1.3 pg/ml, *p* = .009), and t400t (6.7 ± 1.26 pg/ml, *p* = .006) groups were significantly lower that the levels in the DOX (35.6 ± 2.11 pg/ml) group. On the other hand, serum estradiol level was significantly higher in the DOX compared with the normal group (13.26 ± 1.19 pg/ml, *p* = .009, Figure 6c).

### 3.6 Gene expression

**FIGURE 7:**
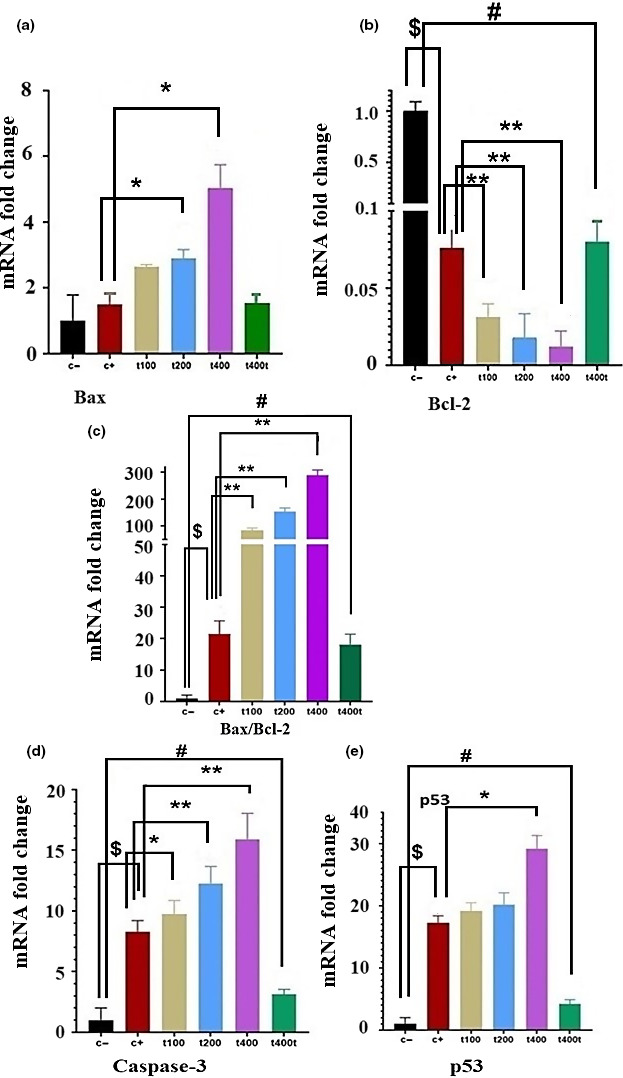
The effect of *Trifolium pratense* on (a) Bax, (b) Bcl‐2, (c) caspase‐3 and d. p53 gene expression of tumors tissue in negative control (C−), positive control (doxorubicin [DOX]), the extract groups (t100, t200 and t400) and t400t (*M* ± *SD*; mean difference between groups compared by using one‐way ANOVA test). ^$^(*p* < .05) statistically significant between DOX and negative control group, ^#^(*p* < .05) statistically significant between t400t and negative control group, *(*p* < .05) and **(*p* < .01) statistically significant between treatment and DOX group

To evaluate apoptosis in tumor tissues, the mRNA expressions of apoptotic genes (caspase‐3, p53, Bax, and Bcl‐2) were measured. The administration of DOX significantly down‐regulated antiapoptotic Bcl‐2 (*p* = .011) and up‐regulated pro‐apoptotic caspase‐3 (*p* = .009) and p53 (*p* = .003) compared with the C− group. The co‐administration of DOX + 100 mg/kg of the plant extract significantly up‐regulated the mRNA expression of caspase‐3 (*p* = .042) and down‐regulated Bcl‐2 (*p* = .008) compared with the DOX group. In the DOX + 200 mg/kg group, the mRNA levels of caspase‐3 (*p* = .006) and Bax (*p* = .039) were significantly elevated while that of Bcl‐2 (*p* = .002) was significantly down‐regulated in comparison with the C− group. Finally, the administration of 400 mg/kg of the *T. pratense* extract in combination with DOX significantly up‐regulated the mRNA expressions of caspase‐3 (*p* = .006), p53 (*p* = .016), and Bax (*p* = .011) and down‐regulated Bcl‐2 (*p* = .0006) mRNA level compared with the DOX group. In addition, at the dose of 400 mg/kg, there were significant up‐regulations of p53 (*p* = .029) and caspase‐3 (*p* = .039) and down‐regulation of Bcl‐2 (*p* = .026) compared with the C− group (Figure 7).

### 3.7 Tumor expressions (%) of p53 and Ki‐67

**FIGURE 8:**
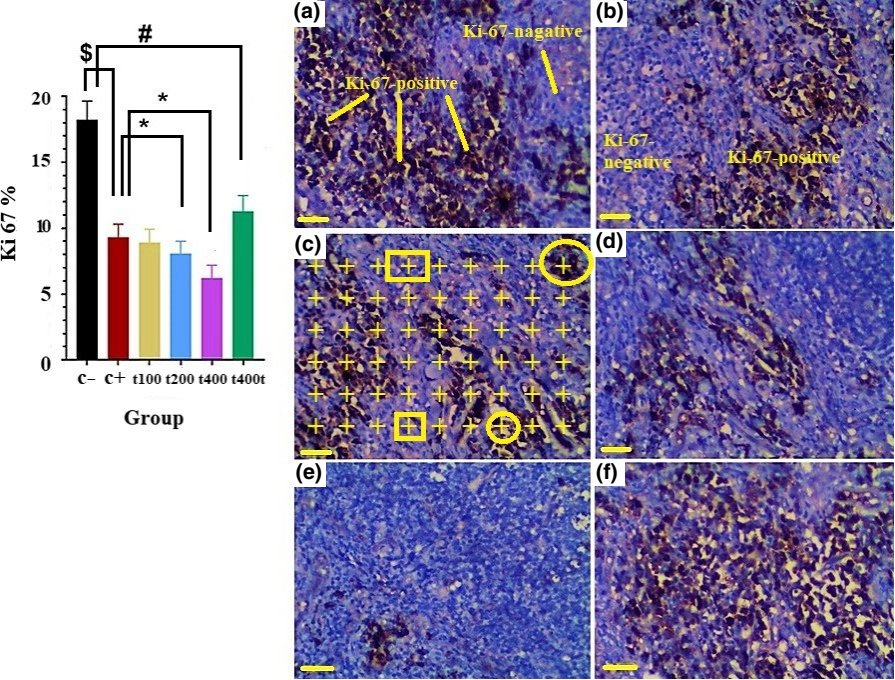
The effect of *Trifolium pratense* on Ki‐67 expression of tumors tissue in negative control (C−, a), positive control (doxorubicin [DOX], b), the extract groups [t100 (c), t200 (d) and t400 (e)] and t400t (f). The points on the Ki‐67 positive cell (circle)/points on the Ki‐67 negative cell (square) × 100 are counted in each field (10 field/tumor) and reported as (*M* ± *SD*; mean difference between groups compared by using one‐way ANOVA test). ^$^(*p* < .05) statistically significant between DOX and negative control group, ^#^(*p* < .05) statistically significant between t400t and negative control group, *(*p* < .05) statistically significant between treatment and DOX group (scale bar = 30 μm, ×400)

**FIGURE 9:**
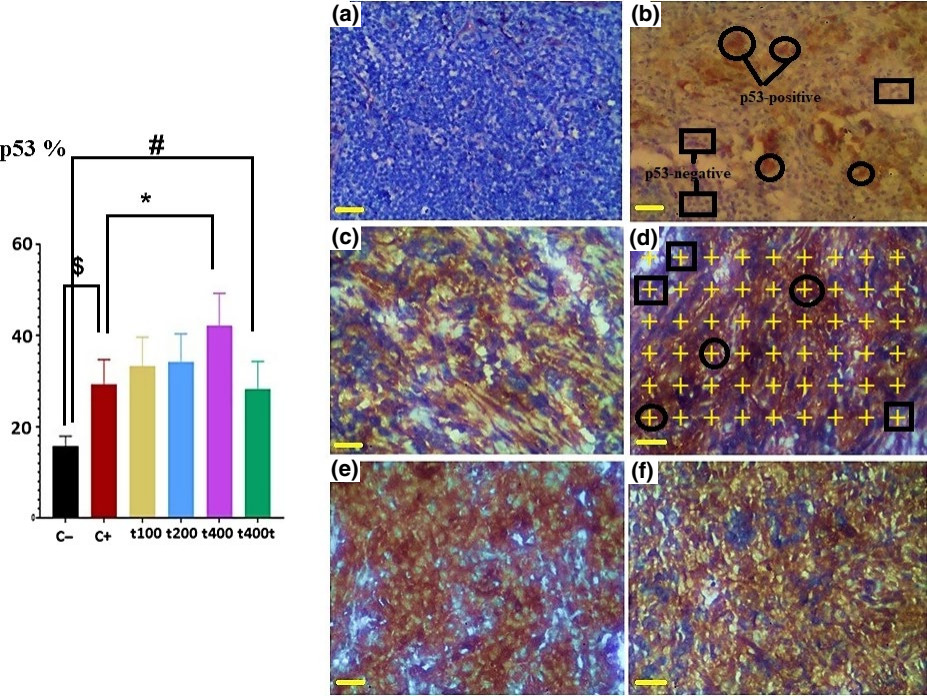
The effect of *Trifolium pratense* on p53 expression of tumors tissue in negative control (C−, a), positive control (doxorubicin [DOX], b) and the extract groups [t100 (c), t200 (d) and t400 (e)] and t400t (f). The points on the p53 positive cell (circle)/points on the p53 negative cell (square) × 100 is counted in each field (10 field/tumor) and reported as (*M* ± *SD*; mean difference between groups compared by using one‐way ANOVA test). ^$^(*p* < .05) statistically significant between DOX and negative control group, ^#^(*p* < .05) statistically significant between t400t and negative control group, *(*p* < .05) statistically significant between treatment and DOX group (scale bar = 30 μm, ×400)

The count of cells expressing phosphorylated Ki‐67 in tumor tissues significantly decreased in the DOX as compared to the C− group (*p* = .029). Also, the expression of this marker was significantly lower in the DOX + 200 mg/kg (*p* = .027) and DOX + 400 mg/kg (*p* = .01) than the DOX group. Treatment with 400 mg/kg of the *T. pratense* extract significantly decreased the ratio of Ki‐67 positive cells as compared to the C− group (*p* = .039, Figure 8). On the other hand, in the DOX (*p* = .029) and DOX + 400 mg/kg (*p* = .011) groups, significant increments were observed in the ratio of p53 positive cells as compared to the C− group. Finally, the ratio of p53 positive cells was significantly higher in the DOX + 400 mg/kg than the DOX group (*p* = .034, Figure 9).

## 4 DISCUSSION

*Trifolium pratense* extract, in synergy with DOX, inhibited the growth of orthotopic TNBC tumors and the proliferation of cancerous cells in 4T1‐tumor bearing BALB/c mice through inducing dose‐dependent apoptosis in vivo. Our data showed that *T. pratense* extract increased the survival of tumor‐bearing mice by augmenting hormonal, inflammatory, and antioxidant pathways against the tumor. DOX is an effective chemotherapeutic agent for treating TNBC, but its use in clinical setting is limited due to the therapeutic (5 mg/kg) and toxicity (8 mg/kg) doses (Mohajeri & Sahebkar, 2018). Today, complementary compounds can be used to reduce the side effects and enhance the anticancer potential of chemotherapeutic drugs.

The combination of red clover extract and DOX synergistically increased the serum levels of IFN‐γ and IL‐12 cytokines compared to the groups treated with either DOX or red clover extract alone. IL‐12 plays a critical role in activating antitumor immunity and inhibiting angiogenesis in vivo and in vitro. The serum levels of this cytokine were found to be decreased in gastrointestinal and breast tumors (Colombo & Trinchieri, 2002).

Isoflavones and their metabolites influence the production of IL‐12 and IFN‐γ by NK cells (Mace et al., 2019). IFN‐γ is a potent activator of macrophages through up‐regulating p53, p21, and p27. Furthermore, IFN‐γ promotes the differentiation of naive CD4^+^ T into Th1 cells, stimulates the expression of MHC molecules presenting tumor antigens, mediates the cell cycle arrest, and increases the apoptosis and susceptibility of tumor cells to immune recognition and destruction. The inhibition of IFN‐γ signaling facilitated tumor development and progression in chemically induced, transplanted, or spontaneously raised breast tumors in mice (Porta et al., 2005). DOX causes cytokine‐mediated systemic inflammation and multiorgan damages in cancer patients. This drug can also stimulate the immune system by directly activating CD8^+^ or γδ T cells leading to an antitumor response through inducing the release of IFN‐γ and IL‐17 by Th_1_ lymphocytes (Galluzzi, Senovilla, Zitvogel, & Kroemer, 2012). Phytoestrogens have a dual effect on the production of IL‐12 and IFN‐γ. Genistein, by binding to ERα, was shown to increase IFN‐γ expression and augment Th_1_ responses (Kuiper et al., 1998). This isoflavone increased IFN‐γ production by cytotoxic T and Th_1_ cells in female DO11.10 mice and mouse models of cervical cancer (Ghaemi et al., 2012; Mohammadi, Zangeneh, Zangeneh, & Haghighi, 2020). However, Zhang *et al*. showed that daidzein and genistein decreased NK cell activity and IFN‐γ production (Zhang, Song, Cunnick, Murphy, & Hendrich, 1999). In another study, autocrine IFN‐γ increased the mRNA expression of ERβ and enhanced the sensitivity of MCF‐7 BC cells to tamoxifen (NIu et al., 2015). Also, long‐term estrogen treatment increased IFN‐γ at mRNA and protein levels in splenic and thymic lymphocytes of normal C57BL/6 mice (Karpuzoglu‐Sahin, Zhi‐Jun, Lengi, Sriranganathan, & Ahmed, 2001). Mostafa et al. (2014) showed that estradiol (E_2_), ERα‐triggered signaling, and estrogen analogs such as tamoxifen and phytoestrogens regulated the production of IFN‐γ in ER^+^ (MCF‐7 and BT‐474) and even ER^−^ (SK‐BR‐3 and MDA‐MB‐231) BC cell lines (Mostafa et al., 2014). The results of these studies were in line with ours. Actually, red clover extract contains isoflavones which can role as estrogen analogs increasing IFN‐γ production in 4T1‐tumor bearing mice.

ROS, by mediating various signal transduction pathways, can play a major role in tumor development, survival, and metastasis (Ma et al., 2020). ROS can induce the activation and synthesis of AP‐1 and regulate cell growth, proliferation, and apoptosis by affecting the levels of transcription factors such as p53. Although p53 can act as antioxidant to eliminate free radicals and ensure cell survival, this molecule can trigger cellular death in response to extreme oxidative stresses (Liu, Chen, & Clair, 2008).

In addition, the exposition of mammalian carcinoma cells to hydrogen peroxide enhanced lung metastasis in mice. This fact shows that ROS, via weakening the attachment of tumor cells to the basal lamina or increasing the activity of proteins that regulate cellular motility, can facilitate the distant seeding of metastatic tumor cells (Ahmeda, Zangeneh, & Zangeneh, 2020; Kundu, Zhang, & Fulton, 1995). During early development of tumors, ROS‐induced hypoxia activates signaling pathways which can positively regulate proliferation and angiogenesis. Consistently, in vivo studies showed that the administration of H_2_O_2_ or oxidant drugs such as DOX activated angiogenesis (Monte, Davel, & Lustig, 1997).

According to the results of this study, the extract of red clover increased TAC and GPx activity augmenting the clearance of free radicals. By scavenging free radicals and enhancing the expression of p53 and other apoptotic genes, the constituents of this plant can reduce tumorigenesis and protect the viability of healthy cells against oxidative damage. In our previous study, we found that *T. pratense* extract inhibited the proliferation of MCF‐7 and MDA‐MB‐231 BC cell lines and induced apoptosis and autophagy via activating genes such as p53, Bax, Caspase‐3, LC‐3, BECN‐1, and ATG‐7 in these cell lines (Khazaei & Pazhouhi, 2019).

The p53, as a tumor suppressor protein, increases the expression of pro‐apoptotic Bax and reduces the expression of antiapoptotic Bcl‐2 genes. In the mitochondrial pathway of apoptosis, the Bax/Bcl‐2 ratio controls the expression of caspase‐3 and finally the cell fate (Handayani, Sakinah, Nallappan, & Pihie, 2007). In a previous unpublished study on the compounds and minerals of red clover by LC‐ESI/MS and ICP‐ESI/SEM‐EDS, we found the main constituents of the plant as genistein, biohanin A, formononetin, quercetin, daidzein, luteolin, kaempferol, apigenin, epigallocatechin 3‐gallate polyphenols (flavonoids and isoflavonoids), and elements such as zinc, cobalt, selenium, manganese, and iron (Akbari Bazm et al., 2020). The flavonoids and isoflavones of this plant promote their anticancer effects via arresting the cell cycle at G1 or G2/M phases, inducing apoptosis, inhibiting ROS formation, metabolizing enzymes, and suppressing mediators such as vascular endothelial and basic fibroblast growth factors (Park et al., 2019). In our study, red clover extract, both alone and in synergy with DOX, increased the ratios of p53, Bax/Bcl‐2, and caspase‐3 in dose‐ and time‐dependent manners.

Isoflavones decrease the serum levels of highly active endogenous estrogen by blocking the binding sites of ERs, inhibiting the transactivation of estrogen‐responsive genes by 17β‐estradiol (E2), and suppressing the 17 β‐oxidoreduction of estrogens by 17 β‐HSOR (17 β‐hydroxysteroid oxidoreductases), a type 1 enzyme inhibitor (Mäkelä et al., 1998). Endogenous estrogens boost the growth and proliferation of TNBC and other types of BC tumor cells. Antagonistic isoflavones (e.g., drugs such as tamoxifen and raloxifene) suppress the production and prevent the effects of endogenous estrogens (Messina & Wood, 2008).

Our results also demonstrated that isoflavones of red clover prevented tumor‐induced elevation of serum estrogen in 4T1‐tumor bearing mice. Studies have shown that isoflavones preferentially bind to and transactivate ERβ rather than ERα, which induces conformational changes in the receptor. Austin et al. (2018) showed that ERβ could be targeted by isoflavone compounds in TNBC tumors (Austin et al., 2018). In a randomized placebo‐controlled clinical trial on postmenopausal women, Palomares et al. (2004) showed that isoflavone containing tablets significantly reduced Ki‐67 proliferation marker in breast tumor biopsies (Palomares et al., 2004).

In order to assess the synergistic effect of *T. pratense* extract and DOX in promoting apoptosis, IHC staining was conducted for Ki‐67 and p53 markers. The Ki‐67 and p53 are important prognostic indicators in human BC and have been used to predict response rates to hormonal and chemotherapeutic agents. Based on the Ki‐67 index, BC tumors can be categorized into groups with >44%, 25%–44%, 16%–24%, and <15% activity (Pan, Yuan, Liu, & Wei, 2017). Wood, Register, Franke, Anthony, and Cline (2006) showed that isoflavones decreased serum E_2_ level and lobular proliferation (Ki‐67 expression) in the breasts of ovariectomized adult female cynomolgus monkeys (Wood et al., 2006).

Studies showed that overexpressed p53 was associated with poor prognostic markers such as advanced histopathological grades and high expressions of epidermal growth factor receptor and Ki‐67. Based on the p53 index, the number of positive cells can range from 22% (good prognosis with appropriate response to treatment) to more than 50% (poor prognosis with inappropriate response to treatment; Jacquemier et al., 1994). Patients with TNBC have higher sensitivity to anthracycline‐based chemotherapeutics and show higher pathologic complete response rates (evidenced by the number of Ki‐67 positive cells in IHC) than patients with luminal tumors (Carey et al., 2007). Our study also showed that *T. pratense* and DOX synergistically and dose‐dependently reduced the number of Ki‐67 positive while increased p53 positive cells in BC tumor tissues.

## 5 CONCLUSION

In conclusion, *T. pratense* extract, as a dietary supplement, demonstrated synergistic antitumor effects with DOX, as a conventional chemotherapeutic agent, in TNBC‐challenged mice. *T. pratense* extract delayed the formation of BC tumors and increased apoptosis in 4T1 cells by stimulating T‐cell cytokines production (IL‐12 and IFN‐γ), reducing ROS generation by tumor cells, inhibiting 17‐β‐estradiol (E_2_) synthesis, and finally extending the survival of tumor‐bearing mice. Further investigations are needed to determine the clinical efficacy and safety of *T. pratense* extract in human, especially in patients with TNBC under treatment with DOX. Also, to obtain therapeutic doses in plasma, the active constituents of *T. pratense* extract need to be purified and commercially produced.

## CONFLICT OF INTEREST

The authors declare that there is no conflict of interest.

## AUTHORS' CONTRIBUTIONS

M.A.B. performed all in vivo and in vitro experiments, analyzed the data and wrote the manuscript. M.K. performed in vivo and in vitro experiment and analyzed the in vivo data. M.R.K. contributed to concept and design, and final approval of the manuscript. All authors read and approved the final manuscript.

## ETHIC APPROVAL

The overall process of research with animals was performed under the supervision of the Ethics Committee of Kermanshah University of Medical Sciences (Ethic code: IR.KUMS.REC.1398.359) in line with the protocol of the Animal Ethics Committee (NIH Publication 80‐23, 1996).

## CONSENT FOR PUBLICATION

Not applicable.

## Data Availability

All data generated or analyzed during this study are included in this published article.
